# Supplementary material for: Improvement of German Chamomile (Matricaria recutita L.) for Mechanical Harvesting, High Flower Yield and Essential Oil Content Using Physical and Chemical Mutagenesis
Source: Plants (Basel). 2022 Nov 1;11(21):2940. doi: 10.3390/plants11212940 (PMC9653700; doi:10.3390/plants11212940)
Supplement: Supplementary file 1 [file plants-11-02940-s001.zip › plants-1969401-supplementary.pdf]

Supplementary Table S1: The characterization of morphological characteristics for selected mutants i.e. flowers fresh weight, flowers dry weight, number of flowers, plant height, stem diameter, number of branches, days to flowering in M2 generation of Egyptian chamomile ecotypes.

	Mutants	Mutagens	Flowers fresh weight (g)	Flowers dry weight (g)	Number of flowers	Plant height(cm)	Stem diameter (mm)	Number of branches	Days to flowering
**Fayoum**									
Control		-	153	35.9	952	70	12	15	118
Dwarf	F/D 1	300 Gray	179	39.8	887	49	12	13	86
	F/D 2	300 Gray	89.6	17.2	419	51	7	9	86
	F/D 3	NaN3 .001	25.7	6.7	203	54	11	12	115
Semi-dwarf	F/SD1	300Gray	70	15	206	56	7	15	117
	F/SD 2	400 Gray	63.4	16.5	362	55	9	12	90
	F/SD 3	NaN3 .001	127	28	668	57	7	23	115
	F/SD 4	NaN3 .002	260	65.3	1808	55	6	12	85
	F/SD 5	NaN3 .003	93.2	22.6	517	56	12	15	77
Tall	F/T 1	100 Gray	256	65.9	1742	100	13	22	140
	F/T 2	100 Gray	99.4	24.3	617	101	15	12	141
	F/T 3	100 Gray	112	27.1	554	102	12	25	119
	F/T 4	200 Gray	229	57	1535	95	17	23	85
	F/T 5	NaN3 .001	144	27.4	917	97	13	17	86
	T/F 6	NaN3 .003	172	38.6	719	103	15	30	60
Large stem diameter	F/LSD 1	100 Gray	218	49.3	1600	93	18	22	71
	F/LSD 2	200 Gray	288	70.7	1732	80	19	25	135
	F/LSD 3	NaN3 .002	220	49.4	1225	71	21	31	83
High number of branches	F/HNOB 1	100 Gray	188	47.7	1708	80	15	35	86
	F/HNOB 2	100 Gray	271	69.1	1263	80	13	33	86
	F/HNOB 3	200 Gray	91.2	21.7	583	77	15	43	71
	F/HNOB 4	300 Gray	143	31.1	1056	65	15	35	115
	F/HNOB 5	NaN3 .002	105	19.9	713	71	13	33	99
	F/HNOB 6	NaN3 .003	199	44	1337	80	15	35	64
Early flowers	F/EF 1	200 Gray	175	38.1	945	75	13	18	65
	F/EF 2	300 Gray	191	45.1	1079	81	11	19	62
	F/EF 3	400 Gray	312	67.5	1550	77	13	25	63
	F/EF 4	NaN3 .003	185	40.7	1073	75	12	15	66

	F/EF 5	NaN3 0.001	358	75.8	1782	68	11	23	74
	F/EF 6	100	91.3	19.7	692	75	9	12	98
Late flowers	F/LF 1	100 Gray	141	33.6	1194	92	16	20	160
	F/LF 2	200 Gray	205	51.7	1050	109	10	28	170
	F/LF 3	300 Gray	97	23.1	566	55	9	11	168
	F/LF 4	300 Gray	38.8	8.3	198	60	7	19	170
	F/LF 5	300 Gray	165	35.4	942	60	11	27	169
	F/LF 6	400 Gray	307	71.8	1652	85	17	37	160
	F/LF 7	NaN3 .002	64	14.8	356	70	13	18	165
	F/LF 8	NaN3 .003	82.5	17.7	470	93	12	25	160
	F/LF 9	NaN3 .003	126	29.2	676	65	9	22	163
High number of flowers	F/HNOF 1	200 Gray	331	73.7	2121	80	12	17	85
	F/HNOF 2	400 Gray	362	78.2	2288	72	15	25	87
	F/HNOF 3	NaN3 .001	240	109	2737	88	20	37	61
	F/HNOF 4	NaN3 .002	328	75.4	2358	70	26	31	134
	F/HNOF 5	NaN3 .003	581	129	2499	85	15	33	78
**Benysuif**									
Control	-	-	188.7	38.9	941	81	9	17	132
Dwarf	B/D 1	100 Gray	167	33	914	53	9	9	71
	B/ D 2	300 Gray	44.1	10.1	292	50	0	10	80
	B/D 3	400 Gray	105.8	26.2	633	54	13	10	108
Semi-dwarf	B/SD1	200 Gray	119.7	27.7	673	60	12	13	99
	B/SD 2	200 Gray	143.2	30	541	63	15	12	160
	B/SD 3	300 Gray	109.3	23.7	545	62	13	15	139
	B/SD 4	NaN3 .001	152.9	34.2	960	58	13	15	97
Tall	B/T 1	100 Gray	249.1	50.6	1104	100	11	21	120
	B/ T 2	100 Gray	398.5	69.6	1795	93	19	21	80
	B/T 3	200 Gray	451.6	96.4	1687	95	17	25	85
	B/T 4	300 Gray	320.2	77.1	1851	90	12	25	86
Large stem diameter	B/LSD 1	100 Gray	485	109.9	2388	75	20	28	163
	B/LSD 2	NaN3 .003	398.5	100.9	3663	71	22	30	161
High number of branches	B/HNOB 1	100 Gray	245.6	53.6	1248	92	12	35	160
	B/HNOB 2	100 Gray	210	47	1350	103	15	30	83
	B/HNOB 3	400 Gray	147.9	37.5	926	60	17	47	169
	B/HNOB 4	NaN3 .003	398.5	100.9	3663	71	22	30	161

	B/HNOB 5	NaN3 .003	143.4	28.7	646	66	12	43	130
	B/HNOB 6	NaN3 .003	157	35.3	1024	73	15	35	53
Early flowers	B/EF 1	100 Gray	140.1	29.6	507	71	12	13	58
	B/EF 2	100 Gray	145.8	24	808	85	14	23	70
	B/EF 3	100 Gray	293.5	68.6	1619	100	21	22	74
	B/EF 4	200 Gray	313.6	65.6	1724	70	15	13	74
	B/EF 5	200 Gray	216.4	53.2	1054	90	10	15	71
	B/EF 6	200 Gray	217.3	49.3	999	75	15	17	65
	B/EF 7	200 Gray	198.3	45.9	996	65	13	18	67
	B/EF 8	NaN3 .002	258.7	51.7	1348	60	11	25	55
	B/EF 9	NaN3 .003	36.1	8.3	90	71	11	15	65
Late flowers	B/LF 1	100 Gray	112	22.1	433	85	11	12	170
	B/LF 2	100 Gray	527.7	75.6	2579	100	13	25	173
	B/LF 3	400 Gray	156.8	40.5	868	77	11	11	172
	B/LF 4	NaN3 .001	212.9	45.26	969	80	14	22	175
High number of flowers	B/HNOF 1	NaN3 .003	339.4	49.15	2452	73	10	18	160
	B/HNOF 2	100 Gray	479	103.9	2380	70	19	30	165
	B/HNOF 3	200 Gray	369.3	101.5	3118	72	13	15	99
	B/HNOF 4	200 Gray	435.7	88.24	2902	66	13	25	86
	B/HNOF 5	300 Gray	420.9	101.9	2406	73	11	11	86
	B/HNOF 6	NaN3 .002	480.2	79.1	2600	80	16	16	99
	B/HNOF 7	NaN3 .002	446.4	105.1	2450	66	11	15	162
	B/HNOF 8	NaN3 .003	398.5	100.9	3 663	71	22	30	161
	B/HNOF 9	NaN3 .003	356	79.5	2258	80	13	25	88
	B/HNOF 10	NaN3 .003	513	101	2002	70	13	25	53
**Menia**									
Control	-	-	137	38.6	844.6	68.6	9.5	14.7	112
Dwarf	M/D 1	100 Gray	67.9	14.4	229	52	6	8	70
	M/ D 2	NaN3 .001	35.9	9.5	217	41	6	11	99
	M/D 3	NaN3 .002	65	14.8	371	53	12	15	108
Semi-dwarf	M/SD1	100 Gray	156.8	35.81	1018	60	8	17	117
	M/SD 2	200 Gray	24.2	6.1	141	60	6	11	135
	M/SD 3	400 Gray	164.2	48.7	897	61	9	12	99
	M/SD 4	NaN3 .002	357.8	77.1	2380	60	11	12	108

Tall	M/T 1	200 Gray	414.3	51.5	800	95	19	33	151
	M/T 2	NaN3 .001	447.3	98.4	2424	95	12	15	99
	M/T 3	NaN3 .002	226.7	56.45	964	90	11	15	155
	M/T 4	NaN3 .002	401.7	83.1	2235	100	17	27	134
	M/T 5	NaN3 .003	194.7	48.3	1088	92	12	35	153
Large stem diameter	M/LSD 1	NaN3 .002	365.4	84.18	2195	100	49	18	88
	M/LSD 2	NaN3 .003	353.6	82.5	1307	83	34.4	18	66
	M/LSD 3	200 Gray	438	108	1998	80	31	12	84
High number of branches	M/HNOB 1	100 Gray	195.09	43.3	1274	80	9	35	140
	M/HNOB 2	NaN3 .001	376.22	81	2036	77	11	33	99
	M/HNOB 3	200 Gray	286	61.5	1995	72	22	35	65
	M/HNOB 4	400 Gray	305	68.2	1792	65	23	45	62
Early flowers	M/EF 1	300 Gray	341.2	71.2	1861	81	16	12	72
	M/EF 2	400 Gray	311	72.81	1128	70	8	12	68
	M/EF 3	400 Gray	246.03	54.17	886	77	15	12	68
	M/EF 4	NaN3 .001	325.6	73.1	1768	70	13	16	75
	M/EF 5	NaN3 .003	353.6	82.5	1307	83	34.4	18	66
Late flowers	M/LF 1	100 Gray	247.9	57.2	1329	78	12	21	156
	M/LF 2	200 Gray	414.3	51.5	800	95	19	33	155
	M/LF 3	200 Gray	299.6	76	1823	70	16	15	156
	M/LF 4	NaN3 .002	226.7	56.45	964	90	11	15	151
	M/LF 5	NaN3 .002	258.2	62.1	1545	90	19	13	155
High number of flowers	M/HNOF 1	100 Gray	435.78	98.52	2705	85	12	17	99
	M/HNOF 2	100 Gray	326.9	76.3	2500	80	11	15	115
	M/HNOF 3	200 Gray	388.8	94.7	2358	85	19	25	87
	M/HNOF 4	400 Gray	503.6	124.6	3183	75	12	12	100
	M/HNOF 5	NaN3 .001	433.7	95.1	2288	85	14	25	83
	M/HNOF 6	NaN3 .001	701.9	123	4477	75	15	25	98
	M/HNOF 7	NaN3 .002	376.4	92.6	2540	75	8	16	95
	M/HNOF 8	NaN3 .002	357.8	77.1	2380	60	11	12	108
	M/HNOF 9	NaN3 .002	287.9	68.15	2295	80	14	21	96
	M/HNOF 10	300 Gray	343	73.9	2645	70	13	17	71

Supplementary Table S2: Flowers cut efficiency percentage of dwarf, Semi-dwarf, tall, big stem diameter, high number of branches , early flowers ,late flowers and high number of flowers isolated mutants of M2 generation of German chamomile ecotypes.

	Mutants	Flowers cut efficiency %											
		Fayoum				Benysuef				Menia			
		First cut %	Second cut %	Third cut %	Fourth cut %	First cut %	Second cut %	Third cut %	Fourth cut %	First cut %	Second cut %	Third cut %	Fourth cut %
**Control**	-	12	20.3	36.5	31.2	10.5	20.5	23.4	45.6	7.7	24.3	34.5	33.5
Dwarf	D 1	22.3	5.9	16.6	55.2	15.8	10.9	73.3	0	43.6	14.4	24	18
	D 2	21.2	26.2	28.8	23.8	37.6	7.2	31.6	23.6	48.2	4	23	24.8
	D 3	12.3	16.2	44.3	27.2	3.5	6.8	37.1	52.6	6.5	7.4	45.6	40.5
	**Mean**	18.6	16.1	29.9	35.4	18.97	8.3	47.33	25.4	32.77	8.6	30.87	27.77
Semi-dwarf	SD 1	5.8	9.8	36.7	47.7	4	2.2	49	44.8	2.2	3.3	67.7	26.8
	SD 2	17.9	15.6	17.9	48.6	3.8	45	40	11.2	9.2	37.6	53.2	0
	SD 3	25.8	19.3	28.5	26.4	14.8	6	1	78.2	5.1	4	17.3	73.6
	SD 4	10.7	11.5	32.9	44.9	1.7	1	44.3	53	2.3	2.3	40.6	54.8
	SD 5	3.7	14.6	67.4	14.3	-	-	-	-	-	-	-	-
	**Mean**	12.78	14.16	36.68	36.38	6.075	13.55	33.58	46.8	4.7	11.8	44.7	38.8
Tall	T1	2	19	60.4	18.6	3	2	13.5	81.5	1.3	3	43.7	52
	**T2**	3	2.7	26.2	68.1	7	2	8	83	1	5	49.5	44.5
	T3	1.8	2.7	5.2	90.3	9.4	19.3	55.3	16	0.3	0.6	51.8	47.3
	**T4**	8.8	5.8	56	29.4	8.8	15.1	46.4	29.7	1.4	8.4	70.2	20
	T5	1.4	2.4	36	60.2	-	-	-	-	0.1	1.1	50.4	48.4
	T6	16.1	1.9	71.2	10.8	-	-	-	-	-	-	-	-
	**Mean**	3.4	6.52	36.76	53.32	7.05	9.6	30.8	52.55	0.82	3.62	53.12	42.44
Large stem diameter	LSD 1	9.6	25.2	55.6	9.6	1.3	1.6	46.4	50.7	1.9	2.9	58.2	37
	LSD 2	1.2	1	40.8	57	7	2	8	83	17.6	13.5	33.2	35.7
	LSD 3	3.5	1	47	48.5	-	-	-	-	4.3	1.2	47	47.5
	**Mean**	4.767	9.067	47.8	38.37	4.15	1.8	27.2	66.85	7.933	5.867	46.13	40.07
Large stem diameter	HNOB 1	4.8	15	48	32.2	1.3	1.6	46.4	50.7	0.5	9.4	74.5	15.6
	HNOB 2	3	8	33.5	55.5	4.8	4.8	8.8	81.6	0.7	9	52.8	37.5
	HNOB **3**	16.6	10.4	21	52	0.2	0.8	31.4	67.6	3	30	31.8	35.2
	HNOB **4**	4.8	9.5	25	60.7	0.3	1.1	48	50.6	14.6	5.6	22.5	57.3
	HNOB5	1	2	39.5	57.5	2.5	1.7	8.2	87.6	-	-	-	-
	HNOB **6**	6.5	9.5	64	20	2.4	8.4	34	55.2	-	-	-	-
	**Mean**	6.117	9.067	38.5	46.32	1.917	3.067	29.47	65.55	4.7	13.5	45.4	36.4
Early flowers	EF 1	12.3	11.1	68.6	8	25.8	16.2	21.9	36.1	6.8	19.9	36.1	37.2
	EF 2	16.6	7.8	34.3	41.3	9.3	0.2	7	83.5	33.4	10.7	50	5.9
	EF 3	17	19.7	49	14.3	7	2	8	83	20.3	16.9	52.5	10.3
	EF 4	15	11	37	37	14.3	10	65.2	10.5	8.6	4.5	40.7	46.2

	EF 5	5.5	6.5	38	50	29	1	40	30	17.6	13.5	33.2	35.7
	EF 6	5.3	11.8	52.2	30.7	28.8	18.4	37.5	15.3	-	-	-	-
	EF 7	-	-	-	-	10.5	5.6	11.5	72.4	-	-	-	-
	EF 8	-	-	-	-	5.3	2.3	35.2	57.2	-	-	-	-
	EF 9	-	-	-	-	11.1	11.1	67.8	10	-	-	-	-
	**Mean**	11.95	11.32	46.52	30.22	15.68	7.422	32.68	44.22	17.34	13.1	42.5	27.06
Late flowers	LF 1	1.2	8.3	75.5	15	0.2	1.1	6.2	92.5	0.1	0.9	24.8	74.2
	LF **2**	1	4.5	44.5	50	0.3	1.1	38.5	60.1	1.3	3	43.7	52
	LF 3	0.5	2.8	54.4	42.3	0.3	1	26	72.7	0.2	0.6	30.2	69
	LF **4**	5.8	9.7	36.7	47.8	0.1	0.4	45	54.5	0.3	0.6	51.8	47.3
	LF **5**	2	2.5	15	80.5	-	-	-	-	0.1	3.7	58.6	37.6
	LF **6**	0	2.3	44.5	53.2	-	-	-	-	-	-	-	-
	LF **7**	1	2	39.5	57.5	-	-	-	-	-	-	-	-
	LF **8**	1	4.8	63.4	30.8	-	-	-	-	-	-	-	-
	LF **9**	5.5	4.5	38	52	-	-	-	-	-	-	-	-
	**Mean**	2	4.6	45.72	47.68	0.225	0.9	28.93	69.95	0.4	1.76	41.82	56.02
High number of flowers	HNOF 1	6.7	9	64.8	19.5	0.1	5.7	12.6	81.6	0.7	4.7	46.4	48.2
	HNOF 2	6	4.3	56.8	32.9	0.3	1	29.4	69.3	4.6	5.8	51.6	38
	HNOF 3	6.5	9.5	64	20	0.6	2.8	58	38.6	6.4	5.8	42.8	45
	HNOF 4	1.4	4.6	69	25	6	1	51.6	41.4	11.2	10.8	56.4	21.6
	HNOF 5	8.4	0.6	49	42	6.1	14.9	46	33	7.1	8.7	35.6	48.6
	HNOF **6**	-	-	-	-	4.3	3.5	55	37.2	0.7	2.8	39.5	57
	HNOF 7	-	-	-	-	0.2	4	54.8	41	1.7	3.5	33.5	61.3
	HNOF 8	-	-	-	-	0.3	1.1	48	50.6	2.3	2.3	40.6	54.8
	HNOF **9**	-	-	-	-	4.3	2.2	55.3	38.2	1	4	47	48
	HNOF **10**	-	-	-	-	12.5	29.5	8	50	12.8	15.1	37.8	34.3
	**mean**	5.8	5.6	60.72	27.88	3.47	6.57	41.87	48.09	4.85	6.35	43.12	45.68

D=Dwarf & SD=Semi-dwarf & T =Tall & LSD=Large stem diameter & HNOB=High number of branches & EF=Early flowering & LF=Late flowering and HNOF= High number of flowers.
